# Taishan *Pinus massoniana* pollen polysaccharide inhibits subgroup J avian leucosis virus infection by directly blocking virus infection and improving immunity

**DOI:** 10.1038/srep44353

**Published:** 2017-03-13

**Authors:** Cuilian Yu, Kai Wei, Liping Liu, Shifa Yang, Liping Hu, Peng Zhao, Xiuyan Meng, Mingxu Shao, Chuanwen Wang, Lijun Zhu, Hao Zhang, Yang Li, Ruiliang Zhu

**Affiliations:** 1 College of Animal Science and Technology, Shandong Agricultural University, Taian, Shandong, 271000, China; 2 Poultry Institute, Shandong Academy of Agricultural Science, Jinan, Shandong, 250023, China; 3 Shandong Provincial Center for Animal Disease Control and Prevention, Jinan, Shandong, 250022, China; 4 Taishan Polytechnic, Taian, Shandong, 271000, China

## Abstract

Subgroup J avian leucosis virus (ALV-J) generally causes neoplastic diseases, immunosuppression and subsequently increases susceptibility to secondary infection in birds. The spread of ALV-J mainly depends on congenital infection and horizontal contact. Although ALV-J infection causes enormous losses yearly in the poultry industry worldwide, effective measures to control ALV-J remain lacking. In this study, we demonstrated that Taishan *Pinus massoniana* pollen polysaccharide (TPPPS), a natural polysaccharide extracted from Taishan *Pinus massoniana* pollen, can significantly inhibit ALV-J replication *in vitro* by blocking viral adsorption to host cells. Electron microscopy and blocking ELISA tests revealed that TPPPS possibly blocks viral adsorption to host cells by interacting with the glycoprotein 85 protein of ALV-J. Furthermore, we artificially established a congenitally ALV-J-infected chicken model to examine the anti-viral effects of TPPPS *in vivo*. TPPPS significantly inhibited viral shedding and viral loads in immune organs and largely eliminated the immunosuppression caused by congenital ALV-J infection. Additionally, pre-administration of TPPPS obviously reduced the size and delayed the occurrence of tumors induced by acute oncogenic ALV-J infection. This study revealed the prominent effects and feasible mechanisms of TPPPS in inhibiting ALV-J infection, thereby providing a novel prospect to control ALV-J spread.

Avian leucosis virus subgroup J (ALV-J) belongs to the genus *Alpharetrovirus* of the Retroviridae family and generally causes myeloid leucosis, hemangioma, and immunosuppression in broilers and layers^1^. Since the first time ALV-J was isolated from commercial broiler breeds in 1988, this virus has spread to Japan, USA, Argentina, and Israel in the 1990s and then to Malaysia and China in the 2000s. For the last 10 years, ALV-J infection is ubiquitous in nearly all chicken types worldwide, causing inestimable losses^2^,^3^. Chickens acquire ALV-J infection congenitally through virus-carrying embryos, as well as horizontally through contact with infected chickens or fomites. In clinical settings, the congenital transmission of ALV-J generally appears more deleterious than the limited contact transmission^3^,^4^. Moreover, congenital ALV-J infection generally induces innate immune tolerance, which contributes to the latent existence and permanent viral shedding of ALV-J^5^,^6^. To date, no effective vaccines are available because of the intricate genetic sequence and antigenic variability of ALV-J^7^,^8^. Persistent surveillance and elimination of infected chickens remain the routine method to control ALV-J spread^9^. Thus, an effective way to prevent and control ALV-J infection is crucial to reduce the losses caused by ALV-J infection.

Natural plant polysaccharides, which are polymeric carbohydrate molecules composed of long chains of monosaccharide units, demonstrate potential anti-viral activities^10^. For instance, algal polysaccharide and sulfated *Ulva lactuca* polysaccharide inhibit the replication of herpes simplex virus and Japanese encephalitis virus, respectively^11^,^12^. Moreover, the anti-viral activities of polysaccharides from plants, such as *Panax ginseng, Angelica sinensis*, and *Ganoderma lucidum*, may be used through an indirect pathway by activating immune responses^13^,^14^,^15^. In our previous studies, we have extracted Taishan *Pinus massoniana* pollen polysaccharide (TPPPS) and found that TPPPS exerts remarkable immune enhancement effects on animals^16^,^17^. Furthermore, we identified three effective components from TPPPS (denoted as TPPPS1, TPPPS2, and TPPPS3) through column chromatographic isolation and purification, and these three components showed complementary effects on antioxidant activity, immunomodulation, and anti-viral activity against subgroup B ALV (ALV-B) *in vitro*^18^. However, whether the whole TPPPS also effectively inhibits the infection of ALV-J, which is a more infectious and virulent strain than ALV-B, remains unknown.

The focus of the present study was to explore the inhibitory effect and mechanism of TPPPS on ALV-J infection. We examined for the first time the anti-ALV-J activities of TPPPS in cell culture *in vitro* and then investigated the interaction between TPPPS and ALV-J to reveal the probable mechanism that interferes with viral replication. Moreover, we artificially established a congenitally infected chicken model to simulate the natural infection of ALV-J and then assessed the anti-viral effects of TPPPS *in vivo*. The inhibitory effect of TPPPS on tumor development induced by challenging the chickens with the highly oncogenic strain ALV-J was also evaluated.

## Results

### TPPPS inhibits ALV-J replication *in vitro*

Many plant polysaccharides directly inhibit viral replication^12^,^19^,^20^. To examine the anti-viral characteristics of TPPPS against ALV-J, we first determined the viral replication kinetics in DF-1 cells treated with different TPPPS concentrations. Prior to the experiment, the toxicity of TPPPS to DF-1 cells was examined. The results showed that TPPPS at concentrations ranging from 1 μg/mL to 1 mg/mL exerted no significant side effects on cell activity (see Supplementary Fig. S1).

**Figure 1:**
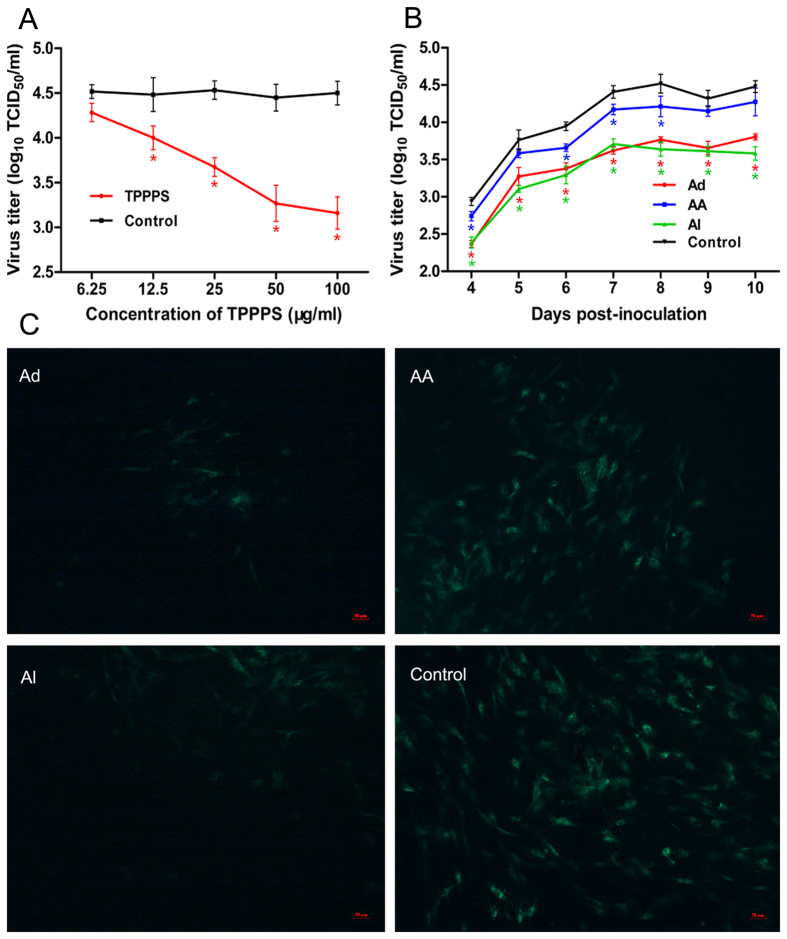
TPPPS inhibits ALV-J replication in DF-1 cells. (**A**) DF-1 cells cultured in 24-well plates were inoculated with 10 μL/well (TCID_50_ of 10^4.75^/mL) of ALV-J. After 10 days of culture in MM containing different TPPPS concentrations, the virus in the supernatants was titrated using ELISA. (**B**) DF-1 cells infected with 10 μL/well (TCID_50_ of 10^4.75^/mL) of ALV-J were treated with 50 μg/mL of TPPPS at different viral infection phases: TPPPS treatment at the adsorption phase (Ad); TPPPS treatment after adsorption (AA); and TPPPS treatment always (Al) during the entire infection process. Supernatants were collected at 24-hour intervals and titrated by ELISA. (**C**) IFA detection on ALV-J-infected DF-1 cells treated with TPPPS 8 days after inoculation. PBS-treated cell wells served as the control. All values shown are the means ± SD of three independent experiments. An asterisk indicates that the value of the corresponding group was significantly different from that of the control group (*P* < 0.05).

Figure 1A shows that the viral titers in cell cultures containing 12.5, 25, 50, and 100 μg/mL of TPPPS were significantly lower than those of the control group (without TPPPS treatment) (*P* < 0.05); in addition, 50 and 100 μg/mL of TPPPS showed the highest inhibitory effects.

To further determine the reaction phase of TPPPS in the whole process of viral replication, 50 μg/mL of TPPPS was chosen to evaluate its antiviral effects at different viral infection phases as described in Materials and Methods. The viral titers in groups Ad and Al were detectable and significantly lower than those in the control group 4 days after inoculation (*P* < 0.05; Fig. 1B). This result indicates that the existence of TPPPS in viral adsorption phase (Ad) and its always existence (Al) in the whole viral growth cycle significantly inhibited ALV-J replication. The viral titers in group AA, which was treated with TPPPS after viral adsorption, were also lower than those in the control group, but their statistical differences were not significant (*P > *0.05). The ALV-J-infected cells in these groups were also illustrated using IFA 8 days after inoculation. The number of infected cells was significantly lower in groups Ad and Al than in groups AA and Control (*P* < 0.05; Fig. 1C). These results imply that TPPPS can remarkably inhibit ALV-J replication *in vitro* and that the inhibitory effect of TPPPS was optimal at the viral adsorption phase (artificially divided).

### TPPPS interferes ALV-J adsorption to host cells

**Figure 2:**
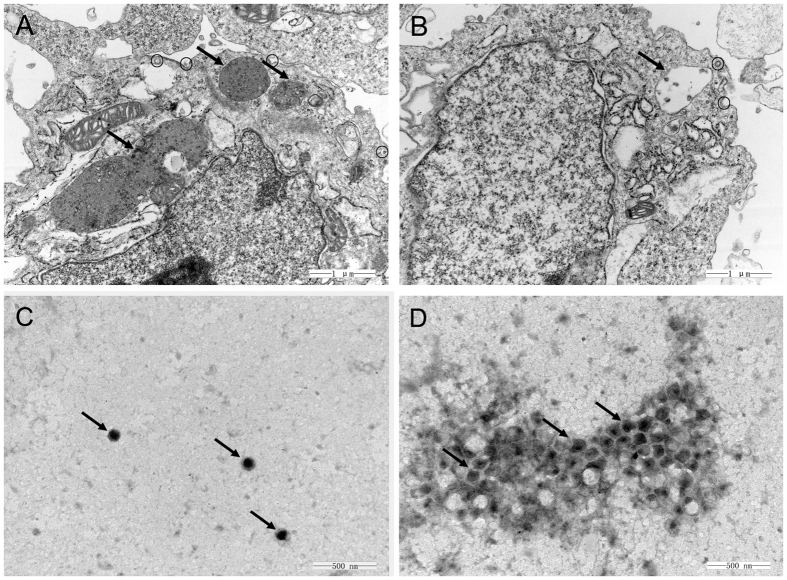
TPPPS interferes with viral adsorption to DF-1 cells. DF-1 monolayers were infected with 10^4.75^ TCID_50_ of ALV-J in the absence (**A**) or presence (**B**) of TPPPS treatment (50 μg/mL). At 4 h post-infection, the cells were centrifuged and fixed to prepare ultrathin sections, and the virions in cells were imaged via TEM (30000×). The virions on the cell membrane surface were denoted by black circles and those in the endosomes were denoted by black arrowheads. The purified ALV-J (**C**) and the mixture of ALV-J and TPPPS (**D**) were imaged via TEM after negative staining (60000×). The virions were denoted by black arrowheads.

One feasible way for the reported plant polysaccharides to inhibit viral infection is to interfere with viral adsorption^12^,^21^. Considering the above results, we determined whether TPPPS inhibits ALV-J infection in this manner. The viral adsorption capability to the DF-1 cells treated with TPPPS was assessed by TEM tomography. Figure 2A shows that several viral particles that are in the phase of membrane fusion were observed on the cell membrane surface of the infected cells without TPPPS treatment; by contrast, a lower number of ALV-J particles was observed on the membrane surface when treated with TPPPS (Fig. 2B). Moreover, more virions in the ALV-J-infected cells were distributed densely in the endosomes (Fig. 2A), whereas an obviously reduced number of virions was found in the endosomes of the TPPPS-treated cells (Fig. 2B). This phenomenon indicates that TPPPS reduced viral adsorption and infection to the cells.

Considering previous findings^19^,^22^, we speculated that TPPPS may act directly on the virus. Thus, we again employed TEM to investigate the interaction between TPPPS and ALV-J particles. The scattered viral particles in the purified virus sample appeared as spheroidal structures with an inner, centrally located electron-dense core, and the average size of the viral particles was approximately 110 nm (Fig. 2C). By contrast, the viral particles treated with TPPPS were found to gather into clusters, and the virions exhibited irregular shapes, low or none electron-density cores, and scattered fragments around the cluster (Fig. 2D), which indicates a direct impact of TPPPS to ALV-J particles.

**Figure 3:**
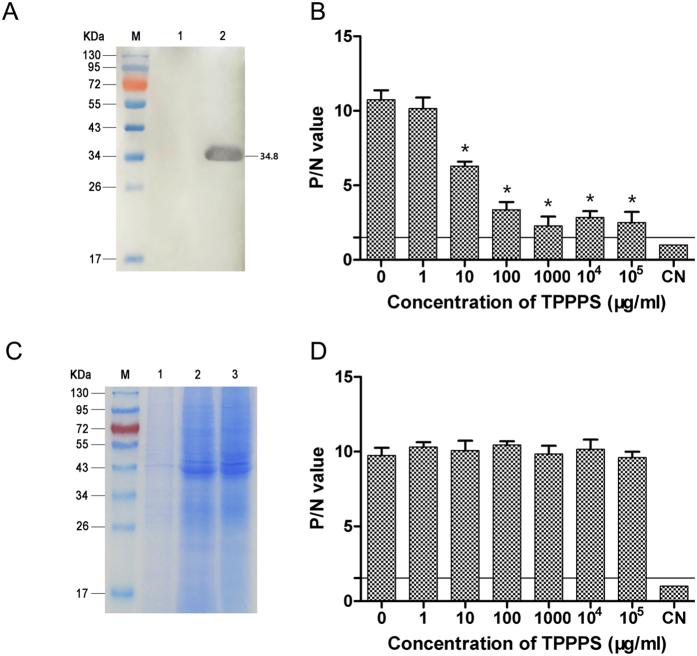
TPPPS blocks the gp85 protein of ALV-J. The gp85 protein of ALV-J was expressed in *Pichia pastoris* and identified through Western blot analysis. M, PageRuler pre-stained protein ladder; lane 1, the culture supernatant of negative *P. pastoris* transformant; lane 2, the purified recombinant gp85 protein (**A**). A blocking ELISA was established on the basis of the expressed gp85 and TPPPS (**B**). The chicken spleen cell membrane proteins were isolated and identified through SDS–PAGE. M, PageRuler pre-stained protein ladder; lane 1, the isolated membrane proteins; lane 2, the residual cytoplasm proteins; lane 3, the whole cell proteins (**C**). The wells coated with the membrane proteins were used as the control for gp85 (**D**). β-actin protein-coated wells served as the negative control (CN) in both ELISAs, and their OD_450_ values were determined. The cut-off point was calculated by mean + 3SD. The P/N value was calculated by the following equation: P/N = Positive mean/Negative mean. All values shown are presented as the means ± SD of three independent experiments. The transverse line in the histogram represents the cut-off point. An asterisk indicates that the value of the corresponding group was significantly different from that of non-TPPPS-treated group (0 μg/ml; *P* < 0.05).

Most virus infections are based on the initial specific binding between viral envelope protein and cell surface receptors. Gp85, the envelope protein of ALV-J, directly binds to the cell surface receptor and then mediates fusion of the viral and cellular membranes^23^. Considering the virustatic effect of TPPPS at early phase of viral infection and its direct impact on virions, we established a blocking ELISA based on the recombinant gp85 expressed in *P. pastoris* (Fig. 3A) and TPPPS as described in Materials and Methods to examine whether there was a direct interaction between them. The wells coated with spleen cell membrane proteins (Fig. 3C) served as the control for those coated with gp85. The P/N values of the wells coated with gp85 significantly decreased with increasing TPPPS amount (blocking agent) (*P* < 0.05; Fig. 3B), whereas those of the wells coated with spleen cell membrane proteins slightly decreased, although the addition of TPPPS reached the highest concentration of 100 mg/mL (*P > *0.05; Fig. 3D). These data indicate that TPPPS interacted with the gp85 protein but not with the cell membrane proteins, implying a possible mechanism on interfering ALV-J adsorption.

### TPPPS reduces viral shedding and viral loads in congenitally ALV-J-infected chickens

To investigate further the practical virustatic effects of TPPPS, we determined the degree of virus propagation in ALV-J-infected chickens with or without TPPPS treatment. Clinically, ALV-J spreads mainly through the congenitally infected embryos to their hatched chickens^3^. Prior to the experiment, we artificially challenged the embryos with ALV-J to induce congenital ALV-J infection and then successively administered the hatched chickens with 5.0 mg/day of TPPPS. Cloacal viral shedding and viral loads in immune organs were determined using the ALV antigen test kit.

**Figure 4:**
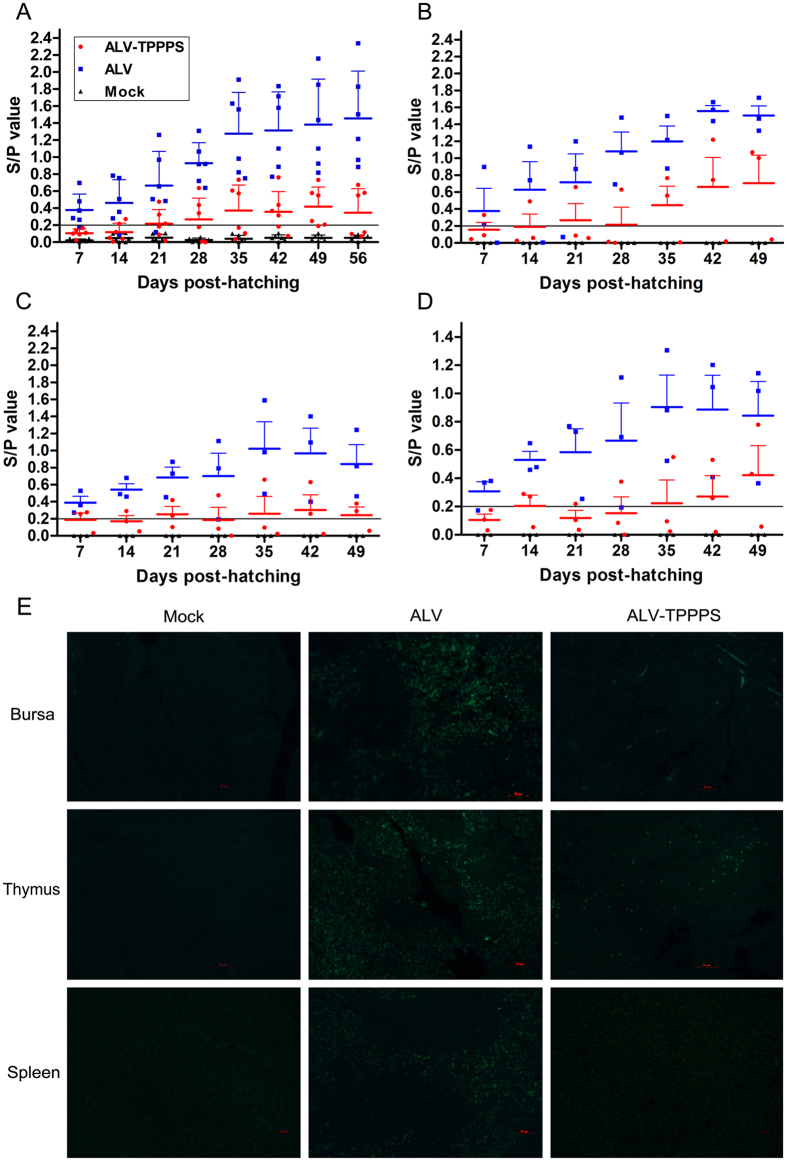
TPPPS reduces viral shedding and viral loads in congenitally ALV-J-infected chickens. Chickens in groups ALV–TPPPS (red) and ALV (blue) were orally administered for 2 weeks with TPPPS and PBS, respectively. Non-infected chickens treated with PBS served as group Mock (black). The cloacal swabs (**A**) of six chickens and the bursa (**B**), thymus (**C**), and spleen (**D**) tissues of three chickens were collected at 7, 14, 21, 28, 35, 42, 49, and 56 dph, and the viral titers were determined by ELISA. S/P value was calculated by the following equation: S/P = (Sample mean − Negative control mean)/(Positive control mean − Negative control mean). The transverse line in the histogram represents the cut-off point. The tissue sections of thymuses, bursas, and spleens from 14-day-old chickens in each group were prepared for viral localization through the immunofluorescence histochemical staining using the ALV-J monoclonal antibody JE9 (200×; **E**). The images shown represent three animals from three independent experiments. Scale bar, 50 μm.

Figure 4 shows that all of the six chickens in group ALV continuously shed virus from 14 dph to 56 dph, whereas only one chicken shed virus at 14 dph in group ALV–TPPPS, and no more than four chickens shed virus at 21–56 dph. Moreover, the mean amount of viral shedding in group ALV–TPPPS was near the detection limit at each sampling time, which were approximately threefold lower than that in group ALV (*P* < 0.05; Fig. 4A). Similarly, the viral titers were significantly lower in the three immune organs (bursas, thymus, and spleen) of group ALV–TPPPS than in those of group ALV at all sampling times (*P* < 0.05), although all immune organs in both groups were virus-positive (Fig. 4B–D). Additionally, the viral colonization in the immune organs fixed at 14 dph was visualized through immunofluorescence histochemical staining using anti-gp85 monoclonal antibody and fluorescent secondary antibodies. We observed intense viral antigen staining in all sections of the immune organs of group ALV (Fig. 4E). However, the staining densities were obviously lower in the bursa and thymus sections of group ALV–TPPPS than in those of group ALV, and few positively stained spleen sections were detected. These results indicate that TPPPS treatment remarkably reduced the viral loads *in vivo* and the viral shedding into the environment.

### TPPPS relieves immunosuppression in congenitally ALV-J-infected chickens

Congenital ALV-J infection in the early embryonic developmental stage usually induces innate immune tolerance, and innate virus infection rapidly causes immune organ hypoplasia, immune function decline, and subsequent immune failure, called immunosuppression^4^,^24^. Our previous studies have demonstrated that TPPPS can significantly improve the function of the immune system in animals^16^,^25^. To verify whether TPPPS can ameliorate the immune states of congenitally ALV-J-infected chickens, we evaluated the degree of immune organ development, T-lymphocyte counts, lymphocyte proliferations, cytokine secretion, and antibody induction in the ALV-J-infected chickens administered with TPPPS.

**Figure 5:**
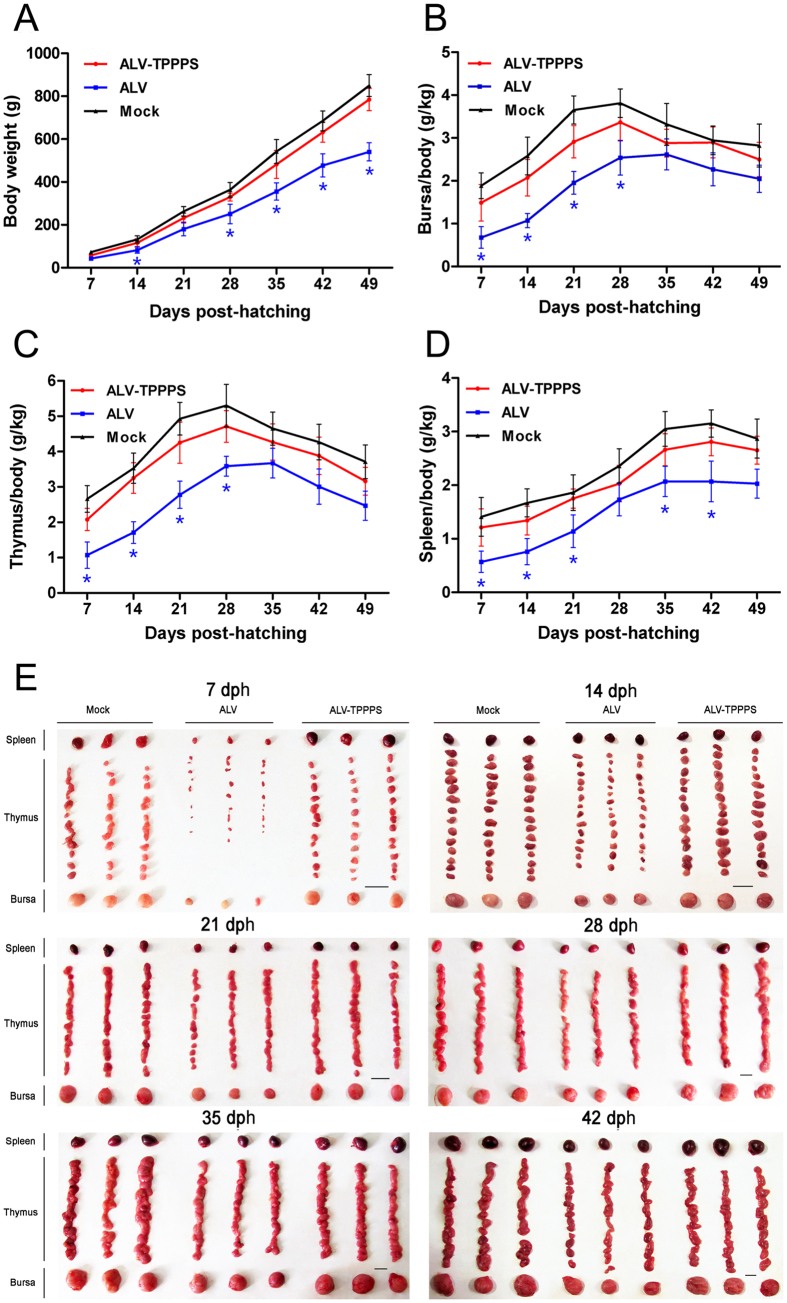
TPPPS affects the body weights and immune organ indexes of congenitally ALV-J-infected chickens. Chickens in groups ALV-TPPPS (red) and ALV (blue) were orally administered for 2 weeks with TPPPS and PBS, respectively. Non-infected chickens treated with PBS served as group Mock (black). The body weights (**A**) and the relative bursa (**B**), thymus (**C**), and spleen (**D**) indexes of three chickens in each group were detected at 7, 14, 21, 28, 35, 42, and 49 dph. All values shown are expressed as means ± SD. An asterisk indicates that the value of the corresponding group was significantly different from that of the control group (*P* < 0.05). The bursas, thymuses, and spleens collected at 7, 14, 21, 28, 35, and 42 dph in each group were also shown (**E**). Scale bar, 1.0 cm.

**Figure 6:**
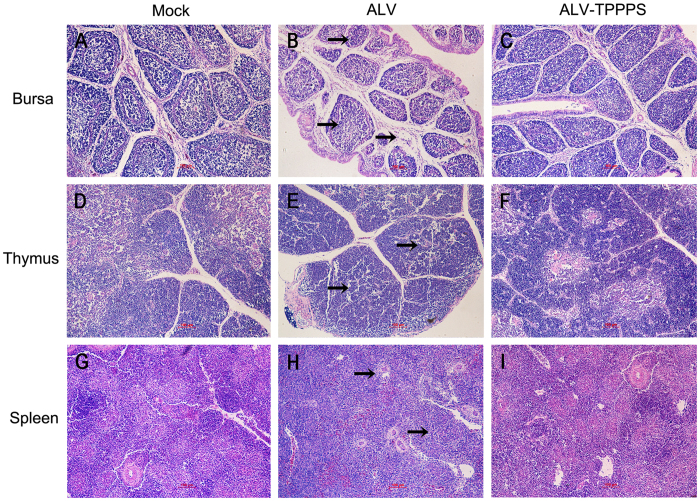
TPPPS treatment relieves the damage in immune organs. Chickens in groups ALV–TPPPS and ALV were orally administered for 2 weeks with TPPPS and PBS, respectively. Non-infected chickens treated with PBS served as group Mock. Histopathological examinations were performed on the thymus, bursa, and spleen from the congenitally infected chickens at 14 dph (H&E staining, 200×). (**A**, **D**, and **G**) Mock-infected bursa, thymus, and spleen showed a normal morphology. (**B**) ALV-J-infected bursa showed lymphoid follicular dysplasia, interstitial expansion, reduced lymphocytes, and loose lymphocyte arrangement in medulla area (black arrows). (**C**) TPPPS-treated bursa showed an almost normal morphology. (**E**) ALV-J-infected thymus showed thymic hypoplasia, reduced lymphocytes, and loose lymphocyte arrangement (black arrows). (**F**) TPPPS-treated thymus showed minor lymphoid depletion. (**H**) ALV-J-infected spleen displayed hypoplasia and obvious white pulp structure (black arrows). (**I**) TPPPS-treated spleen showed a normal morphology. The images shown represent three animals from three independent experiments. Scale bar, 100 μm.

After analyzing the parameters, we found that the growth of the ALV-J-infected chickens (group ALV) was obviously inhibited compared with that of the normal chickens in group Mock (*P* < 0.05; Fig. 5A). By contrast, the growth retardation in the TPPPS-treated chickens (group ALV–TPPPS) was significantly relieved, in which the body weights were even nearly similar to those of the normal chickens. Moreover, severe developmental disorders were observed in the bursas, thymuses, and spleens of group ALV relative to the same organs in group Mock at 7–21 dph; however, the immune organ development in group ALV–TPPPS was significantly better than that in group ALV at 7–21 dph (*P < *0.05) (Fig. 5B–D). Immune organ size and pathological section are presented in Figs 5E and 6. The results intuitively showed an obvious immune organ atrophy and pathological injury in group ALV, especially in the first 2 weeks after hatching. Notably, no remarkable differences in the size and histopathology of immune organs were observed between groups ALV–TPPPS and normal control.

**Figure 7:**
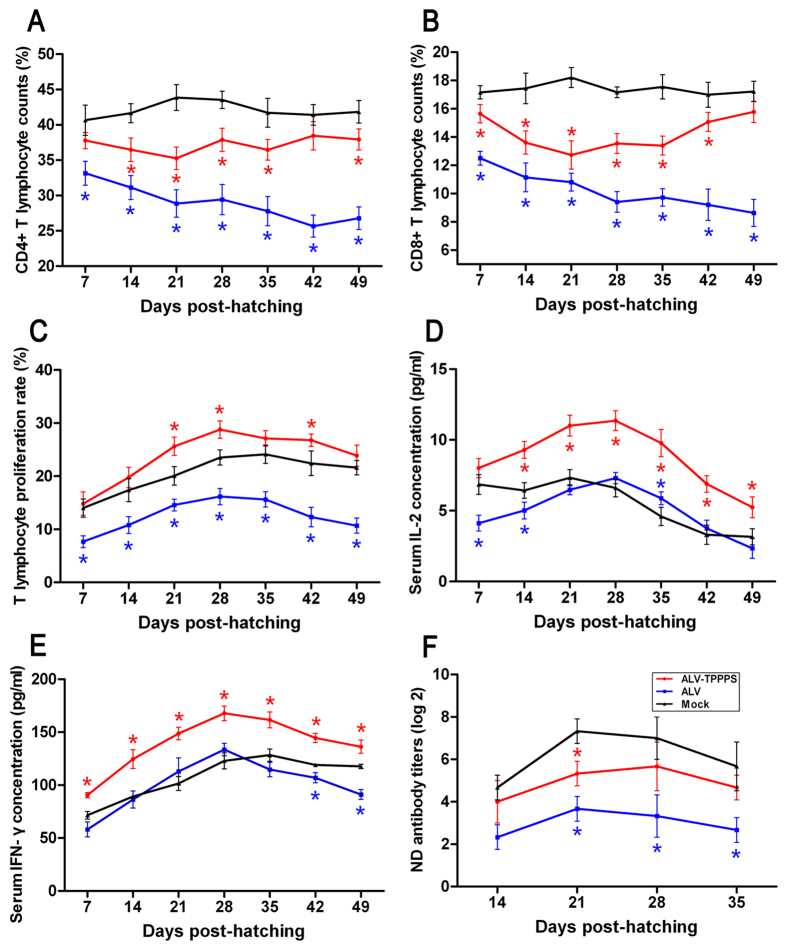
TPPPS affects the immunologic function of congenitally ALV-J-infected chickens. Chickens in groups ALV–TPPPS (red) and ALV (blue) were orally administered for 2 weeks with TPPPS and PBS, respectively. Non-infected chickens treated with PBS served as group Mock (black). The fresh anti-coagulated peripheral bloods and sera were isolated from three chickens in each group at 7, 14, 21, 28, 35, 42, and 49 dph. The percentages of CD4+ (**A**) and CD8+ (**B**) T lymphocytes were detected using flow cytometry. Lymphocyte proliferation (**C**) was detected through the MTT assay. The concentrations of IL-2 (**D**) and IFN-γ (**E**) in sera were determined by ELISA. The anti-ND antibody titers (**F**) were determined by performing the HI test. All values shown are expressed as means ± SD. An asterisk indicates that the value of the corresponding group was significantly different from that of the control group (*P* < 0.05).

In addition to immune organ development, the number and function of lymphocytes, as well as the secretion of cytokines and antibodies, are the key factors used to evaluate the cellular and humoral immunity of organisms. In the present study, the relative populations of CD4+ and CD8+ lymphocytes in peripheral bloods, as well as the lymphocyte proliferation abilities in group ALV, were significantly lower than the normal levels (*P* < 0.05; Fig. 7A–C). These indexes in group ALV–TPPPS reached or even exceeded the normal levels, which were significantly higher than those in group ALV (*P < *0.05). A similar trend was observed in IL-2 and IFN-γ secretions and ND vaccine antibody induction (Fig. 7D–F). These data suggest that the congenital ALV-J infection rapidly induced a distinct immune suppression in the chickens after hatching, especially in the first 2 weeks; however, sustained TPPPS delivery can substantially relieve the impaired immunity.

### TPPPS reduces the pathogenicity of the acute oncogenic Fu-J strain

The acute oncogenic Fu-J virus is a replication-defective virus that sporadically emerges in Chinese chicken flocks. This virus rapidly induces tumors owing to its specific *v-fps* oncogene^26^. To investigate the inhibitory activity of TPPPS against ALV-J infection-induced tumors, we administered TPPPS before and/or after challenging the chickens with tumor leachate containing Fu-J virus as described in Materials and Methods. Then, we monitored the mortality, clinical symptoms, and tumor development daily.

At 6 dpc, 9 out of 20 challenged chickens in group PBS (without TPPPS treatment) showed few tumor nodules, and 7 chickens in group PBS–TPPPS (treated with TPPPS after challenge) showed similar symptoms. However, we did not find any tumor in the chickens in group TPPPS–PBS (treated with TPPPS before challenge) at 6 dpc and in group TPPPS (always treated with TPPPS). Remarkably, all of the 20 chickens in group PBS and 16 chickens in group PBS–TPPPS displayed visible tumors at 9 dpc, whereas only 10 in group TPPPS–PBS and 8 in group TPPPS were found until 12 dpc. From 12 dpc, almost all of the 20 chickens in groups PBS and PBS–TPPPS developed severe tumor and clinical symptoms (swollen heads, dyspnea, and difficulty in eating), and some of the chickens died before 18 dpc. By contrast, the chickens in groups TPPPS–PBS and TPPPS showed substantially smaller tumors (*P* < 0.05) at 12 dpc, and no chickens died at 18 dpc (see Supplementary Fig. S2 and Table 1). Moreover, the normal chickens (group Mock) did not show any symptom (Table 1). These results demonstrated that TPPPS remarkably delayed tumor occurrence and reduced tumor development in the oncogenic ALV-J-infected chickens, although TPPPS cannot completely eliminate tumors. Pre-administration of TPPPS can exert a strengthening tumor-suppressive effect before viral infection.

## Discussion

ALV-J has emerged as a crucial cause of myeloid neoplasia and immunosuppression in chickens. The lack of effective treatment generally leads to serious infection and elimination rates in chicken flocks. The present study explored the inhibitory effect and mechanism of a natural plant polysaccharide, TPPPS, on ALV-J infection. We found that TPPPS can obviously inhibit ALV-J replication by interfering with the viral adsorption in the early phase of infection. Moreover, TPPPS can significantly reduce viral shedding and viral loads in immune organs and can ameliorate immunosuppression in congenitally infected chickens. TPPPS also showed a significant inhibitory effect against tumors induced by the acute oncogenic ALV-J, although TPPPS cannot completely counteract the occurrence of tumors.

Some polysaccharides isolated from plants have demonstrated significant inhibitory effects against animal viruses^22^,^27^,^28^. We have proven that TPPPS, a polysaccharide extracted from *P. massoniana* pollen, inhibits ALV-J replication in a dose-dependent manner *in vitro* and that TPPPS pre-treatment in the first hour of virus infection indicates the strongest inhibition. Furthermore, our TEM assay showed that TPPPS directly affected the morphology and distribution of ALV-J particles. We observed that the virions exhibited irregular shapes and scattered fragments after TPPPS treatment. In the study of Chiu *et al*., they observed a possible direct combination between the *U. lactuca* polysaccharides and Japanese encephalitis virus^12^. Although we are not sure whether there is a direct combination between TPPPS and ALV-J particles in our electron micrograph, the morphology and structure change of virions is certainly attributed to TPPPS treatment. Interestingly, we found a peculiar phenomenon wherein the ALV-J particles clustered after interaction with TPPPS. This phenomenon was previously observed in a study on tobacco mosaic virus, which became clustered after interacting with a polysaccharide peptide^29^. These evidences indicate that direct interaction between TPPPS and ALV-J is likely to interfere the viral infection. The following study demonstrated through blocking ELISA test that TPPPS can interact with the viral envelope protein gp85 but not with the cell membrane proteins. Altogether, these results indicates that TPPPS can obviously inhibit ALV-J replication if pre-treated at early phase of viral infection, and a likely mechanism is to interfere the viral adsorption by directly interact with the virions. Nevertheless, whether TPPPS functions by blocking the interaction between gp85 and its cell surface receptor and also plays roles in other processes of virus infection (e.g., entry, uncoating, or budding) need further verification.

Natural transmission of ALV-J occurs through three modes: congenital infection, horizontal contact, and genetic delivery, among which congenital infection plays a dominant role^3^. In the present study, we established a congenital infection model to simulate the natural infection of ALV-J. We found that the hatchability rate still reached 100% when 10^3^ TCID_50_ of ALV-J was challenged into 7-day-old chicken embryos. This phenomenon implies that the clinically infected chicken embryos can hatch successfully because of the relatively low viral infection dose. Subsequently, we found that the hatched chickens demonstrated a continuous viral shedding during the entire monitoring period. Given that the horizontal transmission of ALV-J predominantly occurred in the first few weeks after hatching^3^, the congenitally infected chickens can serve as sources of virus for horizontal transmission and thus play an important role in ALV-J spread. Our findings revealed that successive TPPPS administration remarkably reduced viral shedding to the external environment. Moreover, the initial viral shedding was delayed for approximately 2 weeks, thereby greatly reducing the risk of early horizontal transmission to uninfected chickens. We also found that TPPPS administration significantly reduced the viral loads of the infected chickens *in vivo* as reflected mainly in the immune organs, in which most of the viral loads were close to the minimum detection limit. Colonization of ALV-J in immune organs is related pathogenically to disrupted immune function and immunosuppression^30^. Thus, the persistently low levels of viral infection facilitate viral clearance, thereby maintaining immune system stability and reducing congenital transmission risk.

Owing to the constitutive embryonic expression of the ALV-J *env* gene, the hatched chickens were immunologically tolerant to viral envelope proteins, which allow ALV-J to reproduce freely in the host^31^,^32^,^33^. Continuous viral replication in the incompletely developed immune systems of neonatal chickens seriously impedes the development of immune organs and their functions^34^. This result is consistent with our observations in the congenitally ALV-J-infected chicken models. The chickens displayed a typical immunosuppression, especially in the first 2 weeks after hatching, as characterized by immune organ atrophy, decline in lymphocyte numbers, and attenuation of cytokine production. Although the immunity improved with age, the immunity levels in the infected chickens were always lower than normal during the monitoring period. Notably, successive TPPPS administration significantly ameliorated the immunosuppression in the ALV-J-infected chickens. The immune-regulating activities of many plant polysaccharides have been studied^35^,^36^,^37^. Our previous studies have proven the excellent immune enhancement effects of TPPPS on animals^16^,^38^. The significant immune recovery effects of TPPPS on ALV-J-induced immunosuppression endow this polysaccharide with more prospects. The present data indicated that TPPPS not only can directly inhibit ALV-J infection but can also enhance immunity, thereby assisting viral clearance. Two other typical symptoms of ALV-J infection, namely, growth retardation and vaccination failure, were found in the congenitally infected models. TPPPS treatment significantly improved the growth rates and ND vaccine antibodies of the immunosuppressed chickens, indicating the crucial roles of TPPPS on improving production performance and in reducing the risk of secondary infection.

An acute oncogenic Fu-J virus that can rapidly induce tumors and death has recently sporadically emerged in Chinese chicken flocks. This oncogenic strain is an endogenous replication-defective virus resulting from a specific gene deletion, and its *v-fps* oncogene can be integrated into the host genome with the help of an exogenous virus to induce acute fibrosarcoma^26^. The chickens challenged with this ALV-J expectedly developed palpable tumors only 1 week post-challenge, and severe tumors and death were observed in these chickens 3 weeks post-challenge. Intriguingly, pre-administration of TPPPS reduced the tumor size and delayed the tumor occurrence for approximately 1 week. These results indicate that TPPPS also exerts a significant inhibitory effect against the oncogenic ALV-J-induced tumors, although TPPPS cannot completely counteract tumor formation. However, due to the lack of method to titrate this disabled ALV, and the challenge dosage in this experiment was likely higher than that in field, implying that TPPPS exhibits enhanced therapeutic potential in clinical settings. By contrast, TPPPS post-administration did not achieve similar effects, implying that earlier TPPPS administration can more effectively prevent and control oncogenic ALV-J infection, especially to susceptible flocks.

In conclusion, we evaluated the anti-ALV-J activity of TPPPS, a natural plant polysaccharide extracted from pine pollen. We found that TPPPS could directly interfere ALV-J infection by interacting with the virions. Moreover, in the artificially constructed congenital infection model, TPPPS not only can reduce viral shedding and viral loads in the ALV-J-infected chickens but can also ameliorate ALV-J-infection-induced immunosuppression, which is another mechanism that complements its anti-viral effects. Moreover, pre-administration of TPPPS before infection significantly inhibited tumor development induced by oncogenic ALV-J infection, and the mechanism underlying tumor inhibition is worthy of further study. Altogether, TPPPS performs a favorable anti-ALV-J activity in a complex mechanism and provides a promising possibility for the prevention and control of ALV-J. The production of *P. massoniana* pollen has already been industrialized in China; thus the development of TPPPS exhibits a huge potential in clinical applications.

## Materials and Methods

### Ethics statement

The animal studies were approved by the Animal Care and Use Committee of Shandong Agriculture University (Permit number: 20010510) and performed in accordance with the “Guidelines for Experimental Animals” of the Ministry of Science and Technology (Beijing, China).

### Viruses, cells, and TPPPS

The NX0101 strain was isolated from a parent breeder farm in 2001^39^ and was titrated in the DF-1 cells by determining the TCID_50_^40^. The Fu-J strain was isolated from natural cases of ALV-J-associated acute fibrosarcoma of crossbreed broilers^41^. The DF-1 cells were maintained in Dulbecco’s modified Eagle’s medium (Gibco) containing 10% fetal bovine serum (Gibco) in 5% CO_2_ at 37 °C. TPPPS (the whole polysaccharide) was prepared in our laboratory^16^.

### Anti-viral activities of TPPPS in DF-1 cells

DF-1 cell monolayers grown in 24-well plates were infected with 10 μL/well of ALV-J solution (10^4.75^ TCID_50_/mL), and cultured in maintenance medium (MM) containing different TPPPS concentrations (6.25, 12.5, 25, 50, and 100 μg/mL). After 1 h of viral adsorption at 37 °C, the supernatants in the wells were replaced with 1.0 mL of maintenance medium (MM) containing corresponding TPPPS concentrations. After 10 days of incubation, viral titers were determined using the ALV P27 antigen test kit (IDEXX, USA).

To investigate the action phase of TPPPS in the viral infection process, DF-1 cells grown on 24-well plates were inoculated with 10 μL/well (10^4.75^ TCID_50_/mL) of ALV-J and then treated with TPPPS at a final concentration of 50 μg/mL at the following artificially divided viral infection phases:

Adsorption (Ad): The cells were exposed to 100 μL of MM containing virus and TPPPS for 1 h at 4 °C. After removal of the supernatant, the cells were washed twice and recovered with pure MM.

After adsorption (AA): The cells were infected with ALV-J in the absence of TPPPS. After viral adsorption for 1 h at 4 °C, the non-adherent viruses were removed. The cells were washed twice and subsequently incubated with MM containing TPPPS.

Always (Al): The cells were infected with ALV-J containing TPPPS. After 1 h of incubation at 4 °C, the supernatants were removed, and the cells were washed and recovered with MM containing TPPPS.

All supernatants in cell culture wells were collected at each 24 h interval and then titrated using the ALV P27 antigen test kit. At 8 d post-inoculation, indirect immunofluorescence assay (IFA) was performed to identify the ALV-J-infected cells by using the ALV-J monoclonal antibody JE9. PBS-treated cell culture wells served as the control.

### Transmission electron microscopy (TEM) assay

DF-1 cell monolayers were infected with 10^4.75^ TCID_50_ of ALV-J with or without TPPPS (50 μg/mL). The monolayers were scraped at 4 h post-infection and then successively fixed in 2.5% glutaraldehyde and 1% osmium tetroxide at 4 °C. The samples were dehydrated in a graded acetone series prior to infiltration and embedding. Ultrathin (50 nm, LKB-V) longitudinal sections were prepared after the location in semi-thin section, stained with uranyl acetate and lead citrate, and then examined under a JEOL-1200EX electron microscope (JEOL, Japan).

The ALV-J solution was concentrated through sucrose density gradient centrifugation. The isovolumetric virus solution (0.55 mg/mL) and TPPPS (50 μg/mL) were mixed and then incubated for 1 h. The mixture was subsequently placed on carbon-coated grids and then negatively stained with 0.01 mL of 2% phosphotungstic acid for 1 min. After washing and drying, the samples were examined under a JEOL-1200EX electron microscope.

### Blocking ELISA

Prior to the experiment, glycoprotein 85 (gp85), the envelope protein of ALV-J, was expressed in the *Pichia pastoris* expression system as previously described^42^. In brief, the gene fragment of gp85 was amplified and cloned into the expression vector pET30a and then transformed into competent *P. pastoris* to obtain the transformant pET30a-gp85. The expressed protein in *P. pastoris* was purified using nickel-chelate chromatography. A blocking ELISA was then established on the basis of the expressed gp85 and TPPPS. The reaction conditions were optimized as follows by using the orthogonal method^43^. The membrane proteins of chicken spleen cells were isolated using membrane protein extraction kit (BestBio, Shanghai) and used as the control for gp85. Rabbit anti-chicken spleen cell membrane protein antibody was prepared from rabbit sera through multiple inoculations. Wells coated with β-actin served as the negative control in both ELISAs; their OD_450_ values were determined, and then the cut-off point was calculated as means + 3SD. The results were expressed as P/N values, which were calculated using the following equation: P/N = Positive mean/Negative mean.

### Establishment of a congenitally ALV-J-infected chicken model

Seven-day-old specific pathogen-free (SPF) chicken embryos were incubated at 37.8 °C and 50–60% relative humidity. A total of 100 embryos were evenly divided into five groups, which were challenged with 0.1 mL of serially diluted virus liquids containing viral loads of 10^0^ to 10^4^ TCID_50_. After hatching, the maximum challenge dose that allowed nearly 100% survival of the chickens was 10^3^ TCID_50_. Two independent tests were conducted to ensure the accuracy of this dose. A total of 120 SPF embryos were then challenged with the same dose to establish a congenitally infected chicken model, and 60 other embryos that were challenged with 0.1 mL of PBS comprised the control group.

### Animal experiment

The 120 1-day-old ALV-J congenitally infected chickens were randomly assigned into two sterilized isolators, denoted as groups I and II. The 60 other 1-day-old normal chickens, denoted as group III, were assigned into another independent isolator. Each chicken in group I was orally administered with 5.0 mg/day of TPPPS for 2 weeks starting from the first day post-hatching (dph). By contrast, each chicken in groups II and III was orally administered with 0.2 mL of PBS daily. At 9 dph, the chickens were intranasally inoculated with attenuated Newcastle Disease (ND) vaccine. Groups I, II, and III were named as ALV-TPPPS, ALV, and Mock, respectively.

### Immune index detection

At 7, 14, 21, 28, 35, 42, and 49 dph, three chickens selected randomly from each group were weighed and euthanized. The immune organs, namely, thymus, bursa, and spleen, were immediately excised surgically and weighed. The immune organ index was calculated using the following equation: Index (g/kg) = (weight of immune organ)/(body weight). The counts of CD4+ and CD8+ T lymphocytes and the lymphocyte transformations in peripheral bloods were determined with previous method^25^. The contents of IL-2 and IFN-γ in the blood samples were detected using chicken IL-2 and IFN-γ ELISA kits (Langdun Bio-technology Co., Ltd., Shanghai). The anti-ND antibody titers in serum samples collected from each group at 14, 21, 28, and 35 dph were determined by the hemagglutination inhibition (HI) test^44^.

### Detection of viral shedding and viral loads in immune organs

Cloacal swabs were randomly collected from six chickens per group at each sampling time point and was dissolved in 1.0 mL of PBS. The collected thymus, bursas, and spleens were finely ground with stroke-physiological saline solution at 1:10 (weight: volume). The viral titers in swabs and tissues were determined using the ALV p27 antigen test kit. The relative amount of antigen in each sample was determined using the following equation: S/P = (Sample mean − Negative control mean)/(Positive control mean − Negative control mean). Viral localization in tissues was also performed to observe the distribution of ALV-J-infected cells in the tissue sections through immunofluorescence histochemical staining using the ALV-J monoclonal antibody JE9.

### Challenge with the acute oncogenic Fu-J strain

A total of 80 1-day-old SPF chickens were randomly assigned into four sterilized isolators and denoted as groups I, II, III, and IV. Ten other chickens were assigned in one independent isolator to serve as the normal control (group V). All 7-day-old chickens in groups I–IV were subcutaneously challenged with 0.2 mL of the cell-free filtrate of acute fibrosarcomas containing the ALV-J Fu-J strain to induce acute fibrosarcomas artificially. Each chicken in group I was orally administered with 5.0 mg/day of TPPPS for 7 consecutive days before the challenge. By contrast, the chickens in group II were administered with TPPPS in the same manner after being challenged. The chickens in group III were administered with the same dose of TPPPS during the entire monitoring period. Groups IV and V were administered with 0.2 mL of PBS for 7 consecutive days. Groups I–V were then denoted as TPPPS–PBS, PBS–TPPPS, TPPPS, PBS, and Mock, respectively.

The clinical status of the sarcoma tissues was monitored continuously after the challenge. The severity of tumors in all chickens of each group were evaluated at 3, 6, 9, 12, 15, and 18 days post-challenge (dpc). At 12 dpc, three chickens in each group were dissected to determine the size and weight of the sarcoma tissues. Tumor severity was examined and scored on the basis of the tumor incidence rates or tumor sizes by using the following scale: 0, no visible changes; 1+, exiguous tumor granulations; 2+, visible intumescence; 3+, visible exceeding intumescence; and 4+, visible exceeding intumescence and tumor ulcerate.

### Statistical analysis

The data were expressed as mean ± SD, and SPSS 17.0 software was used for statistical evaluation. Duncan’s multiple-range test was used to determine the differences among the groups. Statistical significance was considered at *P* < 0.05.

## Additional Information

**How to cite this article**: Yu, C. *et al*. Taishan *Pinus massoniana* pollen polysaccharide inhibits subgroup J avian leucosis virus infection by directly blocking virus infection and improving immunity. *Sci. Rep.* **7**, 44353; doi: 10.1038/srep44353 (2017).

**Publisher's note:** Springer Nature remains neutral with regard to jurisdictional claims in published maps and institutional affiliations.

## Supplementary Material

Supplementary Dataset 1

## Figures and Tables

**Table 1:** Inhibitory effect of TPPPS on tumor development in chickens challenged with acute oncogenic ALV-J.

Group	Days post-challenge											
	3		6		9		12		15		18	
	Tumor positive No./Total No.	severity of tumors^a^	Tumor positive No./Total No.	severity of tumors^a^	Tumor positive No./Total No.	severity of tumors^a^	Tumor positive No./Total No. (weight)(g)^b^	severity of tumors^a^	Tumor positive No./Total No.^b^	severity of tumors^a^	Tumor positive No./Total No.	severity of tumors^a^
TPPPS	0/20	−	0/20	−	0/20	−	8/20 (0.84 ± 0.11^A^)	+	13/17	++	17/17	++
TPPPS–PBS	0/20	−	0/20	−	0/20	−	10/20 (1.25 ± 0.26^A^)	+	14/17	++	17/17	++
PBS–TPPPS	0/20	−	7/20	+	16/20	++	19/20 (4.07 ± 0.23^B^)	+++	17/17	++++	10/10^c^	++++
PBS	0/20	−	9/20	+	20/20	++	20/20 (4.35 ± 0.16^B^)	+++	13/13^c^	++++	7/7^c^	++++
Mock	0/10	−	0/10	−	0/10	−	0/10 (0)	−	0/10	−	0/10	−

^a^Tumor severity was scored on the basis of the incidence rate and mean size of tumors through observation and examination using the following scales: −, no visible changes; 1+, exiguous tumor granulations; 2+, visible intumescence; 3+, visible exceeding intumescence; 4+, visible exceeding intumescence and tumor ulcerate.

^b^At 12 dpc, three chickens in each group were dissected to compare the weight of the sarcoma tissues. The weight was expressed as mean ± SD. Different capital letter superscripts indicate a significant difference (*P* < 0.05).

^c^Reduction of the total No. represents the chickens that naturally died before examination.
