# Supplementary material for: Taishan Pinus massoniana pollen polysaccharide inhibits subgroup J avian leucosis virus infection by directly blocking virus infection and improving immunity
Source: Sci Rep. 2017 Mar 13;7:44353. doi: 10.1038/srep44353 (PMC5347021; doi:10.1038/srep44353)
Supplement: Supplementary Dataset 1 [file srep44353-s1.doc]

**Taishan *Pinus massoniana* pollen polysaccharide inhibits subgroup J avian leucosis virus infection by directly blocking virus infection and improving immunity**

Cuilian Yu^1†^, Kai Wei^1†^, Liping Liu^1^, Shifa Yang^2^, Liping Hu^3^, Peng Zhao^1^, Xiuyan Meng^4^, Mingxu Shao^1^, Chuanwen Wang^1^, Lijun Zhu^1^, Hao Zhang^1^, Yang Li^1^, and Ruiliang Zhu^1^*

^1^ College of Animal Science and Technology, Shandong Agricultural University, Taian, Shandong, 271000, China; ^2^ Poultry Institute, Shandong Academy of Agricultural Science, Jinan, Shandong, 250023, China; ^3^ Shandong Provincial Center for Animal Disease Control and Prevention, Jinan, Shandong, 250022, China; 4 Taishan Polytechnic, Taian, Shandong, 271000, China.

* Corresponding author. College of Animal Science and Technology, Shandong Agricultural University, No. 61 Daizong Avenue, Taian, Shandong, 271018, China.

Tel: +86 538 8242341; Fax: +86 538 8242202. E-mail: [zhurl@sdau.edu.cn](mailto:zhurl@sdau.edu.cn)

^†^ Cuilian Yu and Kai Wei contributed equally to this work.


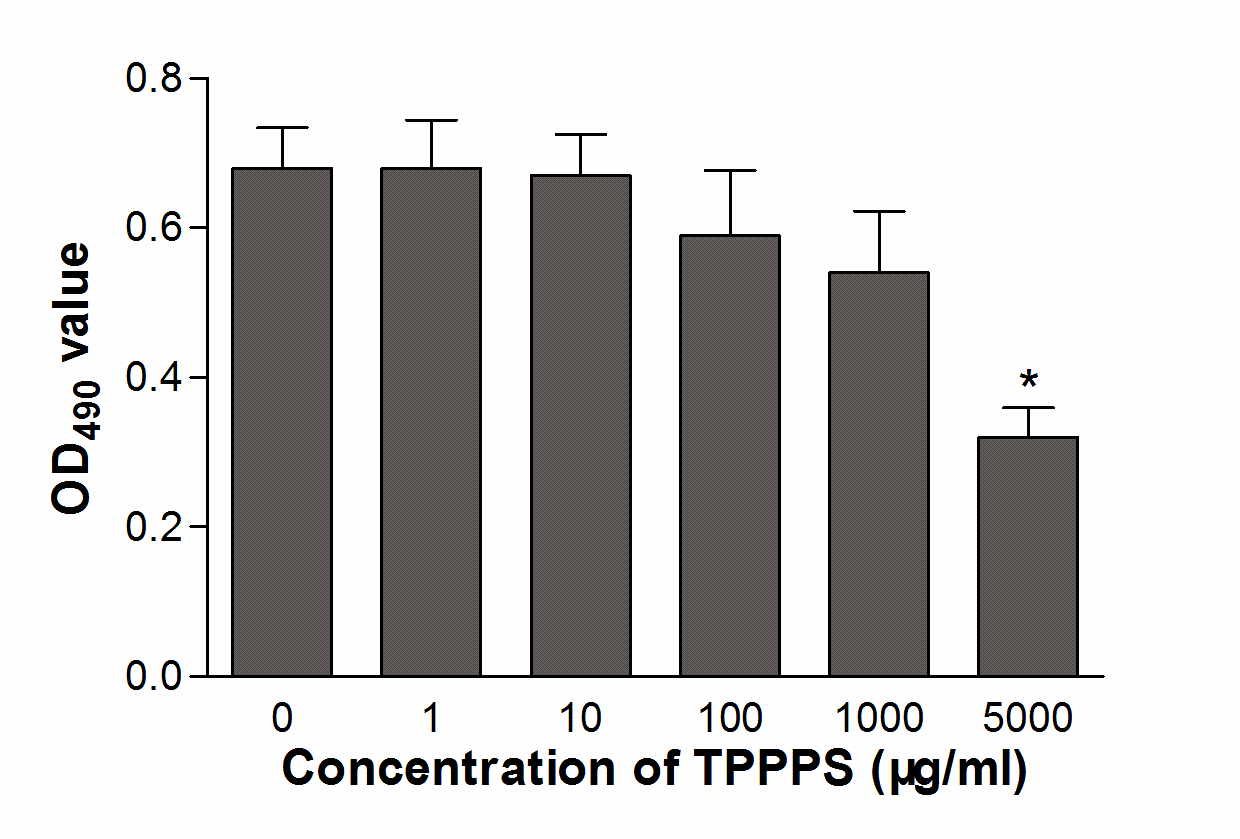
**Supplementary Figures**

**Fig. S1**

**FIG S1 Cytotoxicity test of TPPPS.** DF-1 cells cultured in 24-well plates were inoculated with different TPPPS concentrations for 48 h. After ConA stimulation, the relative viability of DF-1 cells were determined by MTT method, and shown by optical density (OD) at 490 nm. The values are presented as means ± SD from five independent experiments. An asterisk indicates that the value of the corresponding group was significantly different from that of non-TPPPS-treated group (0 μg/ml; *P* < 0.05).


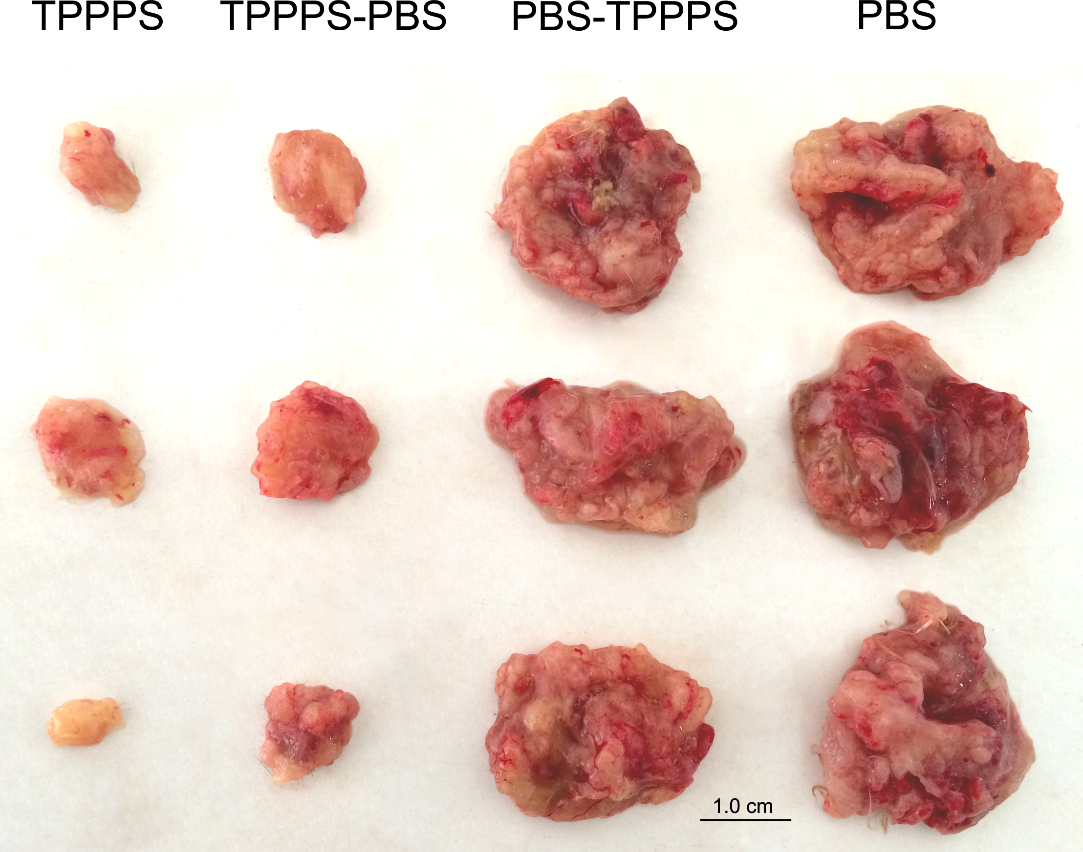
**Fig. S2**

**FIG S2 TPPPS treatment inhibits the development of tumors induced by acute oncogenic ALV-J.** A total of 20 7-day-old chickens in each group were subcutaneously challenged with 0.2 mL of fibrosarcoma filtrate containing Fu-J virus to induce acute fibrosarcomas artificially. The group name TPPPS indicates that each chicken was orally administered with 5.0 mg/day of TPPPS during the entire monitoring period. Groups TPPPS–PBS and PBS–TPPPS comprise chickens administered with the same dose of TPPPS for 7 consecutive days before and after challenge. Group PBS comprises chickens orally administered with 0.2 mL of PBS during the entire monitoring period. At 12 dpc, three chickens in each group were euthanized to compare the size of the sarcoma tissues. Three typical representatives were selected from each group.
